# Impact of longitudinal status change in metabolic syndrome defined by two different criteria on new onset of type 2 diabetes in a general Japanese population: the Tanno–Sobetsu Study

**DOI:** 10.1186/s13098-016-0182-0

**Published:** 2016-09-05

**Authors:** Hirofumi Ohnishi, Shigeyuki Saitoh, Hiroshi Akasaka, Tetsuaki Furukawa, Mitsuru Mori, Tetsuji Miura

**Affiliations:** 1 Department of Public Health, Sapporo Medical University School of Medicine, Sapporo, Hokkaido Japan; 2 Department of Cardiovascular, Renal and Metabolic Medicine, Sapporo Medical University School of Medicine, Sapporo, Hokkaido Japan; 3 Department of Basics and Clinical Medicine, Sapporo Medical University School of Health Science, Sapporo, Hokkaido Japan

**Keywords:** Metabolic syndrome, Status change, Type 2 diabetes

## Abstract

**Background:**

We investigated the effect of longitudinal status change in metabolic syndrome (MetS) assessed by two different criteria on new onset of type 2 diabetes (NODM) in a Japanese population.

**Methods:**

The participants were 827 non-diabetic citizens who had undergone medical examinations in 1994 and 2003 or 2004. We used two different criteria of MetS: the interim criteria by 6 institutions (MetS-INT) and Japanese criteria (MetS-JP). According to the interim criteria, individuals defined as having MetS have any three of the following five components of MetS: obesity, hyperglycemia, high blood pressure, hypertriglyceridemia and low HDL cholesterolemia. According to the Japanese criteria, individuals defined as having MetS must have abdominal obesity plus any two of the following three factors; hyperglycemia, high blood pressure and dyslipidemia (hypertriglyceridemia and/or low HDL-cholesterolemia). According to the status change in MetS, participants were divided into four groups: a non-MetS to non-MetS group, a non-MetS to MetS group, a MetS to non-MetS group and a MetS to MetS group. We calculated odds ratios of these four groups for NODM.

**Results:**

NODM occurred in 65 participants. The odds ratios for NODM were 4.86, 4.97 and 7.50 in the non-MetS-INT to MetS-INT group, MetS-INT to non-MetS-INT group and MetS-INT to MetS-INT group, respectively. On the other hand, the odds ratios were 4.28 and 15.55 in the non-MetS-JP to MetS-JP group and MetS-JP to MetS-JP group, respectively. After adjustment for high fasting plasma glucose at baseline, magnitude of the odds ratio of MetS to MetS group was larger in the Japanese criteria group than in the interim criteria group.

**Conclusions:**

Determining the status of MetS routinely and paying attention to status change in MetS may be important for prevention of type 2 diabetes. MetS defined by the criteria which includes obesity as a prerequisite component may be a stronger risk for type 2 diabetes than MetS defined by the criteria which includes obesity as one of components.

## Background

Metabolic syndrome (MetS) is one of the useful predictors for future occurrence of type 2 diabetes as well as cardiovascular disease [1–8]. However, there are different criteria for diagnosis of MetS, which cause some confusion because there are some differences in prevalence of MetS by using various criteria. A joint interim statement has been issued by the International Diabetes Federation (IDF) Task Force on Epidemiology and Prevention; National Heart, Lung, and Blood Institute (NHLBI); American Heart Association (AHA); World Heart Federation; International Atherosclerosis Society; and International Association for the Study of Obesity. According to the statement, abdominal obesity should not be a prerequisite for diagnosis, but it is 1 of 5 criteria, 3 of which constitute a diagnosis of metabolic syndrome [9]. On the other hand, according to the Japanese criteria [10], as in the previous IDF criteria [11], abdominal obesity is a prerequisite for diagnosis of MetS because there is an emphasis in the Japanese criteria that abdominal obesity is a background for risk accumulation in the pathological mechanism of metabolic syndrome. Because obesity, especially abdominal obesity, is considered to be a strong risk factor for new onset of type 2 diabetes (NODM) [12, 13], there may be some difference in the predictive power for NODM if abdominal obesity is prerequisite or not.

To prevent occurrence of type 2 diabetes and cardiovascular disease, life style intervention is important for individuals with MetS [14, 15]. A recent large-scale observational study showed that life style intervention in individuals with MetS was related with 25 % risk-reduction of type 2 diabetes and 50 % risk-reduction of cardiovascular disease [15]. During life style intervention period, repeated assessment of whether they satisfy MetS criteria or not is important for determining the effectiveness of the life style intervention [16]. Although many studies have shown that MetS at baseline is a useful predictor of the future occurrence of type 2 diabetes, the impact of longitudinal status change in MetS on new onset of type 2 diabetes has not been determined.

In this study, we therefore investigated the effect of longitudinal status change in MetS defined by two different criteria on NODM in a general Japanese population.

## Methods

### Participants

We have been conducting a cohort study called “The Tanno–Sobetsu study” since 1977. Annual health checkups, including standard blood and urine tests and an electrocardiogram, have been conducted for all residents aged 30 years or more of Tanno town and Sobetsu town. The two towns are in rural areas of Hokkaido, the northernmost island of Japan, and the major industry in both towns is agriculture. Most of participants in this cohort are middle-aged and elderly people and their life style, prevalence of obesity, blood pressure, and blood glucose and serum lipid levels are similar to the results of a national survey in Japan. Therefore, this cohort is considered to represent Japanese middle-aged and elderly population.

In this study, of 1908 residents who were aged 30 years or older when they received medical examinations in the two towns in 1994, 938 also underwent medical examinations in 2003 or 2004. From the 938 residents, we excluded 70 individuals without data for MetS components and 41 individuals who had type 2 diabetes in 1994 (individuals with fasting plasma glucose (FPG) ≥126 mg/dl [17] and/or individuals on medication for type 2 diabetes). The remaining 827 individuals were participants in this study.

This study was approved by the Ethics Committee of Sapporo Medical University and we received written informed consent from all participants.

### Measurement items

Waist circumference, body mass index (BMI), systolic blood pressure (SBP), diastolic blood pressure (DBP), fasting plasma glucose (FPG), total cholesterol (T.chol), triglycerides (TG), and HDL cholesterol (HDL-C) were measured in each participant. After 5 min of rest, blood pressure levels (SBP and DBP) were measured twice in a sitting position and average values were used for analysis. FPG level, T.chol level, TG level and HDL-C level were measured by the hexokinase method, cholesterol oxidase method, enzymatic method and the direct method, respectively.

### Criteria of metabolic syndrome

Participants with MetS were determined according to two different criteria: the interim criteria by 6 institutions [9] and the Japanese criteria [10]. Differences among the two criteria are simply described below.

According to the interim criteria, individuals defined as having MetS (MetS-INT) have any three of the following five components of MetS: elevated waist circumference (waist circumference ≥90 cm for men and ≥80 cm for women; Asian criteria), hypertriglyceridemia (TG ≥150 mg/dl and/or on drug treatment for elevated triglycerides), low HDL cholesterolemia (HDL-C <40 mg/dl for men and <50 mg/ml for women), high blood pressure (SBP ≥130 mmHg and/or DBP ≥85 mmHg and/or treatment for previously diagnosed hypertension) and high FPG (FPG ≥100 mg/dl).

According to the Japanese criteria, individuals defined as having MetS (MetS-JP) must have abdominal obesity (waist circumference ≥85 cm for men and ≥90 cm for women) plus any two of the following three factors: dyslipidemia (TG ≥150 mg/dl and/or HDL-C <40 mg/dl and/or specific treatment for these), high blood pressure (SBP ≥130 mmHg and/or DBP ≥85 mmHg and/or treatment for previously diagnosed hypertension) and high FPG (FPG ≥110 mg/dl) (Table 1).

**Table 1:** Comparisons of international criteria of metabolic syndrome and Japanese criteria of metabolic syndrome

	International criteria	Japanese criteria
Absolutely required	None	Central obesity (waist circumference ≥85 cm for men and ≥90 cm for women
Criteria	Any three of the five criteria below	Obesity, plus two of the three criteria below
Obesity	Elevated waist circumference (waist circumference ≥90 cm for men and ≥80 cm for women; Asian criteria)	(Central obesity already required)
High blood pressure	SBP ≥130 mmHg and/or DBP ≥85 mmHg and/or treatment for previously diagnosed hypertension	SBP ≥130 mmHg and/or DBP ≥85 mmHg and/or treatment for previously diagnosed hypertension
Dyslipidemia	TG ≥150 mg/dl and/or on drug treatment for elevated triglycerides	TG ≥150 mg/dl and/or HDL-C <40 mg/dl for both gender and/or specific treatment for these
	HDL-C <40 mg/dl for men and <50 mg/dl for women	
Hyperglycemia	FPG ≥100 mg/dl	FPG ≥110 mg/dl

*SBP* systolic blood pressure, *DBP* diastolic blood pressure, *TG* triglycerides, *HDL-C* HDL cholesterol, *FPG* fasting plasma glucose

### Statistical analysis

According to the status change in MetS between 1994 and 2003 or 2004, participants were divided by using each of the criteria separately into four groups: a non-MetS to non-MetS group, a non-MetS to MetS group, a MetS to non-MetS group and a MetS to MetS group. We calculated the odds ratio of MetS at baseline and the odds ratios of the above four groups for NODM (individuals with FPG ≥126 mg/dl [17] or individuals who were on medication for type 2 diabetes on the basis of the 2003 or 2004 medical examination data).

IBM SPSS Statistics ver.22 was used for statistical analysis. The significance level in all analyses was set at p < 0.05. All numerical values are expressed as mean ± SD or medians and ranges. Student’s t test, analysis of variance (ANOVA), the Kruskal–Wallis test, Dunnett’s test and Fisher’s exact test with Bonferroni correction were used for examination of intergroup differences compared with the non-MetS to non-MetS group and for frequency comparison. Multiple logistic regression analysis was used to estimate the odds ratios of NODM. Age, sex were selected as confounding factors in model 1. Then high FPG (FPG ≥100 mg/dl for MetS-INT and ≥110 mg/dl for MetS-JP) at baseline was additionally included in the Model 2 (Model 2).

## Results

Table 2 shows the baseline characteristics of participants by gender. Mean age, waist circumference, TG, FPG and percentages of participants with abdominal obesity assessed by the Japanese criteria, smoking, IFG, high TG, low HDL-C assessed by the Japanese criteria and MetS-JP were significantly higher in men than in women. SBP, T.chol, HDL-C and percentage of participants with low HDL-C assessed by the interim criteria were significantly higher in women than in men. There were no significant differences in BMI, DBP, percentages of participants with a family history of type 2 diabetes, abdominal obesity assessed by the Asian criteria, high blood pressure and MetS-INT between men and women.

**Table 2:** Baseline characteristics of subjects by sex, 1994

	Men	Women	p
n	347	480	
Mean age (years)	59.6 ± 9.0	58.3 ± 8.5	0.034
Family history of type 2 diabetes (%)	9.2	11.3	0.387
Body mass index (kg/m^2^)	23.5 ± 3.1	23.5 ± 2.8	0.784
Waist circumference (cm)	83.2 ± 9.2	75.2 ± 8.1	<0.0001
Abdominal obesity (Asian criteria) (%)	23.3	29.0	0.079
Abdominal obesity (Japanese criteria) (%)	42.1	5.6	<0.0001
Systolic blood pressure (mmHg)	131.3 ± 17.3	134.1 ± 19.2	0.028
Diastolic blood pressure (mmHg)	78.4 ± 9.1	77.7 ± 9.8	0.320
Total cholesterol (mg/dl)	183.8 ± 29.7	199.6 ± 31.6	<0.0001
Triglycerides (mg/dl)	144.2 ± 106.7	117.7 ± 76.6	<0.0001
HDL-C (mg/dl)	53.2 ± 13.5	57.7 ± 13.5	<0.0001
Fasting plasma glucose (mg/dl)	94.8 ± 10.4	93.0 ± 8.8	0.008
Smoking (%)	69.7	7.3	<0.0001
High blood pressure (%)	56.8	58.8	0.617
Impaired fasting glucose (%)	27.4	19.6	0.009
High triglycerides (≥150 mg/dl) (%)	30.3	17.1	<0.0001
Low HDL-C (INT) (<40 mg/dl for men and <50 mg/dl for women) (%)	15.3	27.1	<0.0001
Low HDL-C (JP) (<40 mg/dl for men and women) (%)	15.3	8.8	0.004
MetS-INT (%)	20.7	18.5	0.477
MetS-JP (%)	17.3	1.5	<0.0001

Abdominal obesity by Asian criteria: 90 cm for men and 80 cm for women; Abdominal obesity by Japanese criteria: 85 cm for men and 90 cm for women; *MetS-INT* metabolic syndrome assessed by the interim criteria by 6 institutions, *MetS-JP* metabolic syndrome assessed by the Japanese criteria

**Fig. 1:**
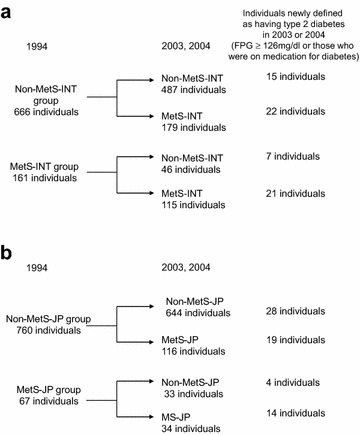
**a** Shows number of individuals newly defined as having type 2 diabetes in four status-change groups when using the interim criteria of MetS. **b** Shows number of individuals newly defined as having type 2 diabetes in four status-change groups when using the Japanese criteria of MetS. *MetS-INT* metabolic syndrome defined by the interim criteria, *MetS-JP* metabolic syndrome defined by the Japanese criteria follow-up results by different criteria of MetS

Figure 1 shows the follow-up results in this study. During the follow-up period, NODM occurred in 65 participants. When using the MetS criteria by 6 institutions, there were 666 individuals in the non-MetS-INT group and 161 in the MetS-INT group at baseline. Of the 666 individuals in the non-MetS-INT group, 487 remained in the non-MetS-INT category in 2003 or 2004, and the remaining 173 individuals changed to the MetS-INT category in 2003 or 2004. One hundred fifteen of the 161 individuals in the MetS-INT group remained in the MetS-INT category in 2003 or 2004, and the remaining 46 individuals changed to the non-MetS-INT category (Fig. 1a). When using the Japanese MetS criteria, there were 760 individuals in the non-MetS-JP group and 67 in the MetS-JP group at baseline. Of the 760 individuals in the non-MetS-JP group, 644 remained in the non-MetS-JP category in 2003 or 2004, and the remaining 116 individuals changed to the MetS-JP category in 2003 or 2004. Thirty-four of the 67 individuals in the MetS-JP group remained in the MetS-JP category in 2003 or 2004, and the remaining 33 individuals changed to the non-MetS-JP category (Fig. 1b).

Table 3 shows the baseline characteristics among status-change categories defined by the interim criteria of MetS. BMI, waist circumference, SBP, DBP, FPG and percentages of participants with abdominal obesity, high BP, high FPG, high TG and low HDL-C were significantly higher in the non-MetS-INT to MetS-INT group, MetS-INT to non-MetS-INT group and MetS-INT to MetS-INT group than in the non-MetS-INT to non-MetS-INT group (reference group). Mean age and T.chol were higher in the non-MetS-INT to MetS-INT group and MetS-INT to MetS-INT group than in the reference group. The percentage of men was lower in the non-MetS-INT to MetS-INT group and was higher in the MetS-INT to non-MetS-INT group than in the reference group. The percentage of participants with a family history of type 2 diabetes was higher in the MetS-INT to MetS-INT group than in the reference group. HDL-C was lower in the above three groups than in the reference group.

**Table 3:** Baseline characteristics among status-change categories defined by international criteria of metabolic syndrome

	Non-MetS-INT to Non-Met-INT	Non-MetS-INT to MetS-INT	MetS-INT to Non-MetS-INT	MetS-INT to MetS-INT	p
n	487	179	46	115	
Mean age (years)	57.8 ± 9.1	59.8 ± 7.8*	60.8 ± 7.5	62.8 ± 7.8*	<0.001^a^
Men (%)	44.8	28.4^#^	65.2^#^	46.5	<0.001^b^
Family history of type 2 diabetes (%)	5.5	6.3	2.2	16.3^#^	0.005^b^
Body mass index (kg/m^2^)	22.5 ± 2.5	24.3 ± 2.7*	25.7 ± 2.1*	27.1 ± 2.4*	<0.001^a^
Waist circumference (cm)	75.7 ± 8.3	79.8 ± 8.5*	91.7 ± 5.2*	89.7 ± 7.5*	<0.001^a^
Systolic blood pressure (mmHg)	128.8 ± 17.4	135.4 ± 17.5*	145.2 ± 16.3*	150.4 ± 16.1*	<0.001^a^
Diastolic blood pressure (mmHg)	76.3 ± 9.1	79.3 ± 9.3*	85.7 ± 9.3*	83.3 ± 9.0*	<0.001^a^
Total cholesterol (mg/dl)	189.9 ± 30.7	196.6 ± 32.6*	194.8 ± 32.9	203.6 ± 33.1*	0.001^a^
Triglycerides (mg/dl)	93 (42–635)	125 (45–554)	141 (52–506)	157 (47–1127)	<0.001^§^
HDL-C (mg/dl)	59.3 ± 13.2	51.5 ± 13.4*	50.6 ± 10.2*	46.7 ± 10.7*	<0.001^a^
Fasting plasma glucose (mg/dl)	91.7 ± 8.9	95.1 ± 8.8*	100.6 ± 8.8*	101.6 ± 10.5*	<0.001^a^
Smoking never smoker (%)	65.3	73.6	50.0	65.1	0.001^b^
Ex-smoker (%)	8.6	5.8	21.7	17.4	
Current smoker (%)	26.1	20.7	28.3	17.4	
Abdominal obesity (Asian criteria) (%)	12.3	27.4^#^	60.9^#^	87.2^#^	<0.001^b^
High blood pressure (%)	45.4	64.9^#^	91.3^#^	94.2^#^	<0.001^b^
High fasting plasma glucose (%)	13.3	23.1^#^	65.2^#^	53.5^#^	<0.001^b^
High triglycerides (%)	10.7	25.5^#^	65.2^#^	60.5^#^	<0.001^b^
Low HDL-C (%)	10.5	30.3^#^	47.8^#^	54.7^#^	<0.001^b^

Asian criteria of abdominal obesity, waist circumference ≥90 cm for men and ≥ 80 cm for women

High blood pressure, systolic blood pressure ≥130 mmHg and/or diastolic blood pressure ≥85 mmHg and/or medication for hypertension

High fasting plasma glucose, fasting plasma glucose ≥100 mg/dl; high triglycerides, triglycerides ≥150 mg/dl; Low HDL-C, HDL cholesterol <40 mg/dl for men and <50 mg/dl for women

Continuous variables are expressed as means ± SD or medians (range)

* p < 0.05 vs. Non-MetS to Non-MetS group using Dunnet’s test

^#^p < 0.05 vs. Non-MetS to Non-MetS group using Bonferroni correction

^a^Analysis of variance (ANOVA)

^b^Fisher’s exact test

^c^Kruskal–Wallis test

Table 4 shows the baseline characteristics among status-change categories defined by the Japanese criteria of MetS. BMI, waist circumference, SBP, DBP, FPG, percentage of men, and percentages of participants with abdominal obesity, high BP, high FPG, high TG and low HDL-C were significantly higher in the non-MetS-JP to MetS-JP group, MetS-JP to non-MetS-JP group and MetS-JP to MetS-JP group than in the non-MetS-JP to non-MetS-JP group (reference group). Mean age was higher in the non-MetS-JP to MetS-JP group and MetS-JP to non-MetS-JP group than in the reference group. T.chol was higher in the non-MetS-INT to MetS-INT group than in the reference group. The percentage of participants with a family history of type 2 diabetes tended to be higher in the MetS-JP to MetS-JP group than in the reference group, but the difference was not statistically significant. HDL-C was lower in the above three groups than in the reference group.

**Table 4:** Baseline characteristics among status-change categories defined by Japanese criteria of MetS

	Non-MetS-JP to non-Met-JP	Non-MetS-JP to MetS-JP	MetS-JP to Non-MetS-JP	MetS-JP to MetS-JP	p
n	644	116	33	34	
Mean age (years)	58.2 ± 8.9	60.9 ± 7.1*	62.2 ± 7.4*	61.2 ± 10.3	0.001^a^
Men (%)	34.9	53.5^#^	93.9^#^	85.3^#^	<0.001^b^
Family history of type 2 diabetes (%)	6.2	6.9	3.0	17.6	0.087^b^
Body mass index (kg/m^2^)	22.9 ± 2.7	25.1 ± 2.8*	25.0 ± 1.9*	27.2 ± 3.3*	<0.001^a^
Waist circumference (cm)	76.1 ± 8.3	84.2 ± 7.4*	89.9 ± 4.3*	94.2 ± 6.5*	<0.001^a^
Systolic blood pressure (mmHg)	130.9 ± 18.2	137.1 ± 17.6*	144.9 ± 15.5*	146.3 ± 16.9*	<0.001^a^
Diastolic blood pressure (mmHg)	77.0 ± 9.5	79.8 ± 8.5*	82.5 ± 7.4*	86.0 ± 8.5*	<0.001^a^
Total cholesterol (mg/dl)	191.7 ± 31.4	200.3 ± 34.3*	192.7 ± 27.8	194.0 ± 31.0	0.064^a^
Triglycerides (mg/dl)	98.5 (42–635)	129.5 (51–1109)	186 (47–506)	181.5 (62–1127)	<0.001^c^
HDL-C (mg/dl)	57.8 ± 13.3	51.4 ± 13.0*	46.0 ± 10.9*	43.6 ± 11.4*	<0.001^a^
Fasting plasma glucose (mg/dl)	92.5 ± 9.0	96.0 ± 9.2*	100.2 ± 10.5*	103.0 ± 11.0*	<0.001^a^
Smoking never smoker (%)	71.9	53.5	39.4	35.3	<0.001^b^
Ex-smoker (%)	7.0	14.7	24.2	26.5	
Current smoker (%)	21.1	31.9	36.4	38.2	
Abdominal obesity (Japanese criteria) (%)	10.3	34.5^#^	100.0^#^	100.0^#^	<0.001^b^
High blood pressure (%)	52.3	67.2^#^	93.9^#^	97.1^#^	<0.001^b^
High fasting plasma glucose (%)	4.0	7.8^#^	30.3^#^	38.2^#^	<0.001^b^
High triglycerides (%)	15.2	32.8^#^	81.8^#^	70.6^#^	<0.001^b^
Low HDL-C (%)	7.8	17.2^#^	30.3^#^	44.1^#^	<0.001^b^

Japanese criteria of abdominal obesity, waist circumference ≥85 cm for men and ≥90 cm for women

High blood pressure, systolic blood pressure ≥130 mmHg and/or diastolic blood pressure ≥85 mmHg and/or medication for hypertension

High fasting plasma glucose, fasting plasma glucose ≥110 mg/dl; High triglycerides, triglycerides ≥150 mg/dl; Low HDL-C, HDL cholesterol <40 mg/dl for both men and women

Continuous variables are expressed as means ± SD or medians (range)

* p < 0.05 vs. Non-MetS to Non-MetS group using Dunnet’s test

^#^p < 0.05 vs. Non-MetS toNon-MetS group using Bonferroni correction

^a^Analysis of variance (ANOVA)

^b^Fisher’s exact test

^c^Kruskal–Wallis test

Table 5 shows the results of multiple logistic regression analysis. The odds ratios for NODM in individuals with MetS-INT and individuals with MetS-JP at baseline adjusted for age and sex were 3.46 and 5.15, respectively (Model 1). When we additionally adjusted for high FPG at baseline (Model 2), MetS-INT lost its significance, but the odds ratio of MetS-JP was 3.45 and still retained its statistical significance. As for status change of MetS, the odds ratios for NODM were 4.86, 4.97 and 7.50 in the non-MetS-INT to MetS-INT group, MetS-INT to non-MetS-INT group and MetS-INT to MetS-INT group (Model 1), respectively. However, in Model 2, only the odds ratio of non-MetS-INT to MetS-INT group and MetS-INT to MetS-INT group retained its significance. On the other hand, the odds ratios were 4.28 and 15.55 in the non-MetS-JP to MetS-JP group and MetS-JP to MetS-JP group, respectively (Model 1), and both of them still retained their significance after adjustment for high FPG at baseline (Model 2). Status change of MetS-JP to non-MetS-JP was not selected as a significant risk for NODM. Magnitude of the odds ratio of MetS to MetS group was larger in the Japanese criteria group than in the interim criteria group.

**Table 5:** Odds ratios of MetS at baseline and longitudinal status change in MetS for new onset of type 2 diabetes

	MetS-INT		MetS-JP	
	Model 1	Model 2^a^	Model 1	Model 2^b^
MetS at baseline	3.46^**^ (2.02–5.91)	1.49 (0.82–2.69)	5.15^**^ (2.60–10.20)	3.45^**^ (1.68–7.05)

	Model 1	Model 2^a^	Model 1	Model 2^b^
Non-MetS to non-MetS	1.00	1.00	1.00	1.00
Non-MetS to MetS	4.86^**^ (2.44–9.70)	4.16^**^ (2.03–8.53)	4.28^**^ (2.27–8.08)	4.11^**^ (2.08–8.13)
MetS to non-MetS	4.97^**^ (1.89–13.07)	1.75 (0.63–4.89)	3.06 (0.95–9.87)	0.93 (0.25–3.48)
MetS to MetS	7.50^**^ (3.67–15.33)	3.38^**^ (1.57–7.25)	15.55^**^ (6.71–36.07)	6.64^**^ (2.46–17.89)

*Model 1* adjusted for age, sex

*Model 2* Model 1 + FPG ≥100 mg/dl at baseline

*Model 3* Model 1 + FPG ≥110 mg/dl at baseline

*MetS-INT* metabolic syndrome assessed by the interim criteria by 6 institutions, *MetS-JP* metabolic syndrome assessed by the Japanese criteria

* p < 0.05

** p < 0.01

## Discussion

The main findings of this study are [1] MetS assessed by both of the criteria at baseline were significant predictors of NODM and MetS-JP only retained its significance after adjustment for high FPG at baseline, [2] status change of non-MetS to MetS was a significant risk for NODM, and maintenance of status of MetS was the strongest risk for NODM, and [3] magnitude of the odds ratio of MetS to MetS group was larger in the Japanese criteria group than in the interim criteria group.

The results of this study showed that MetS-JP still maintained its significance after adjustment for high FPG at baseline. According to the Japanese criteria of MetS [10], the definition of MetS must include abdominal obesity as in the previous International Diabetes Federation (IDF) definition [11], because the accumulation of visceral fat in individuals with MetS is considered to be important for the mechanism underlying the accumulation of risk factors for cardiovascular disease. Abdominal obesity, which is one of the background factors of insulin resistance, may lead to the accumulation of coronary risk factors and to the development of type 2 diabetes. Ford et al. [18] reported that MetS was a risk factor for future occurrence of type 2 diabetes in Europeans and that abdominal obesity and impaired fasting glucose (IFG) were the two strongest predictors of incident diabetes. Marott et al. [19] also reported that waist circumference and glucose level were strongly associated with occurrence of type 2 diabetes. Mukai et al. reported that when using the criteria of metabolic syndrome reported by AHA/NHLBI [20], metabolic syndrome that did not include the IFG component was also a significant risk factor for the development of type 2 diabetes and that the coexistence of metabolic syndrome and IFG greatly increased the risk of future type 2 diabetes in the general Japanese population [7]. Therefore, the Japanese criteria of MetS, which can efficiently identify individuals with the coexistence of abdominal obesity and high FPG, may be a more useful predictor for NODM than the interim criteria.

In this study, new change to MetS was a strong risk for the development of type 2 diabetes and maintenance of the status of MetS was the strongest risk factor for NODM when using both criteria. Status change of MetS may be an indication of the duration of MetS, and the results of this study may mean that the longer the duration of MetS is, the higher is the risk for development of type 2 diabetes. After adjustment for high FPG at baseline, magnitude of odds ratio of MetS to MetS group was larger in the Japanese criteria group than in the interim criteria group. One of the reasons why status change of MetS-JP was a stronger risk for type 2 diabetes than status change of MetS-INT may be the issue of whether abdominal obesity is prerequisite or not. Because abdominal obesity plays an important role in the status change of MetS-JP, status change of MetS-JP to MetS-JP means a continuation of abdominal obesity. On the other hand, status change of MetS-INT to MetS-INT does not always mean continuation of abdominal obesity because abdominal obesity is not a prerequisite for diagnosis in the criteria. Another reason may be that there are several differences in the cut-off points of components of MetS between the criteria. Differences in the cut-off points of waist circumference, high FPG level and HDL-C level may affect the prevalence of MetS and the percentages of men and individuals with higher levels of FPG in the MetS group. When using the Japanese criteria, prevalence of MetS-JP was lower than that of MetS-INT, but percentages of men and individuals with IFG, which is considered to be a risk factor for NODM, were higher in the MetS-JP group than in the MetS-INT group. These may also explain why the results of status change of MetS to non-MetS were different between MetS-INT and MetS-JP.

One of clinical implications of this study is that the results may be useful in education for community dwellers with MetS by public health workers. The impact of maintained status of MetS on NODM may motivate community dwellers with MetS to change their life styles. It is known that life style intervention for individuals with impaired glucose tolerance (IGT) or MetS can prevent type 2 diabetes [21–25]. In the present observational study, we could not find an effect of status change in MetS to non-MetS on prevention of NODM. Further interventional studies are needed to assess the effect of improvement in the status of MetS on NODM.

There are some limitations in this study. First, we could not assess the gender difference in the effect of status change of MetS on NODM because our sample size was relatively small. Further large-scale studies are needed to evaluate the gender difference. Second, there may be a self-selection bias because we included participants who received the health checkups in both 1994 and 2003 or 2004. Therefore, we may have underestimated the impact of status change of MetS on NODM. Third, we could not assess the components of both criteria of MetS for which changes are important to predict NODM and we could not assess the appropriate cut-off points of components of both criteria because of the small sample size. Further studies are needed to examine these issues. Forth, we may underestimate number of new onset type 2 diabetes because we did not conduct 75 g oral glucose tolerance test to define new onset of type 2 diabetes. Fifth, we could not consider effects of several confounding factors such as antihypertensive agents, family history of type 2 diabetes and body mass index in multiple logistic regression models because of small sample size. Further large-scale studies are needed to consider effects of several confounding factors other than fasting plasma glucose level at a time.

## Conclusion

In conclusion, the results of this study show that not only MetS at baseline but also longitudinal status change in MetS are useful for assessing the risk of future development of type 2 diabetes. Our results suggest that routinely determining the status change of MetS and paying attention to status change of MetS may be important for prevention of the future occurrence of type 2 diabetes. Status change of MetS defined by the criteria which includes obesity as a prerequisite component may be a stronger risk for type 2 diabetes than MetS defined by the criteria which includes obesity as one of components.
